# Knowledge, Beliefs, and Psychosocial Effect of Acne Vulgaris among Saudi Acne Patients

**DOI:** 10.1155/2013/929340

**Published:** 2013-12-29

**Authors:** Magdy A. Darwish, Ahmed A. Al-Rubaya

**Affiliations:** ^1^Department of Family and Community Medicine, College of Medicine, University of Dammam, P.O. Box 2114, Dammam 31451, Saudi Arabia; ^2^Postgraduate Center of Family and Community Medicine, Directorate of Health Affairs, Ministry of Health, Eastern Province, Saudi Arabia

## Abstract

*Objective*. This study was conducted to assess the knowledge, beliefs, and psychosocial effect of acne vulgaris among acne patients attending referral dermatology clinic in Al-Khobar city. *Material and Methods*. A cross-sectional study was conducted on all Saudi acne patients (males and females) attending referral dermatology clinic in Al-Khobar Governmental Hospital. The data were collected by using a structured self-administered questionnaire. *Results*. Like other studies conducted before, we found that 58.33% of our sample have poor knowledge about factors that affect acne vulgaris with a significant correlation with both age and gender (*P* = 0.012 and *P* = 0.031, resp.). There was significant association of reporting affected social activities with age and educational level (*P* = 0.023 and *P* = 0.013, resp.). Variation between both genders regarding reporting feeling stressed due to acne was significant (*P* = 0.001). The majority of our sample sought medical advice after one year. The most commonly used treatment for acne vulgaris before seeking medical help was peeling products. The majority of our patients thought that acne needs no treatment by physicians. Doctors' treatment is considered guaranteed and safe by the vast majority of our patients. *Conclusion*. This study showed that knowledge about acne is still insufficient among acne patients.

## 1. Introduction

Acne vulgaris is an extremely common disorder. Prevalence of acne varies among different populations in different studies from 50% [1, 2] to 80% [3–5]. There is general recognition that there are many factors in the etiology of acne vulgaris [6]. Causes could be attributed to both genetic and environmental factors. There is familial predisposition of severe forms of acne that support a genetic component. Acne usually occurs around puberty but it may start late in the thirties and forties (in adulthood) [5, 7, 8]. It takes several years before spontaneous remission [5]. Prognosis of the disease is usually good but, as a chronic disease, relapses even during treatment could occur. It can remit spontaneously [9].

Few studies are interested in exploring knowledge and experiences of acne patients towards acne [10]. In a study that was conducted by Brajac et al. (2004) they found that “Acne was considered as a trivial and transitory condition by 52% of the acne patients and 44% of the family physicians” [11]. Students had misconceptions regarding the causes of acne [2]. Not only the knowledge about acne causes that is lacked, but also natural course and therapy were very low, and it has been found among patients of all ages, physicians, and nurses as well [12]. Individuals have various beliefs and perceptions about what causes acne vulgaris and how it could be managed [3–5, 13]. Despite the high prevalence of acne, there is too much wrong beliefs and deficiencies in the knowledge about it [14]. Some patients think it is a normal phase in the development into adulthood. Others believe that it resolves spontaneously once the affected person gets in early adulthood.

Treatment of acne is usually postponed that patients may wait more than one year before seeking medical advice [15]. Acne is a disorder in which adherence has a major impact on treatment outcome [15]. Improvement of current knowledge and understanding of the different presentations of acne allow for individualization, tailoring treatment, and improved outcomes for acne patients [16].

Psychological and social consequences of acne vulgaris are considerable although it is not causing severe morbidity or physical disability [2, 17–20]. Acne often leads to significant psychological and physical morbidity [21, 22]. More than a cosmetic nuisance, acne can produce anxiety, depression, and other psychological problems that affect patients' lives in ways comparable to life-threatening or disabling diseases [23]. Given the fact that acne causes psychological suffering, acne can affect social, vocational, and academic performance of teenagers [24]. Severe acne may lead to scarring and disfigurement, aggravating the already present psychosocial aspects of this condition [25]. Suicidal ideation was found to be around 6-7% in acne patients [3, 26, 27]. Some studies have shown that there are gender differences in the effect of psychological trauma on acne patients [28, 29]. Psychological comorbidities in acne are probably greater than generally assumed. Therefore, emotional problems due to acne should be taken seriously and included in the treatment plan [23].

This cross-sectional study was conducted to assess knowledge, beliefs, and psychosocial effects of acne among acne vulgaris patients attending dermatology referral clinics, Al-Khobar Governmental Hospital, Al-Khobar, Saudi Arabia.

## 2. Material and Methods

A cross-sectional study was conducted to assess knowledge, beliefs, and psychosocial effects of acne among acne vulgaris patients attending Al-Khobar Governmental Hospital, Dermatology Referral Clinics, Eastern Province, Saudi Arabia. All acne patients (males and females) attending Al-Khobar Governmental Referral Dermatology Clinic during the period from November 2012 to the end of December 2012 were involved. Data were collected using structured, self-administered questionnaire which was designed after reviewing the recent literature and similar questionnaires and based on the objectives of the study putting in consideration sociocultural backgrounds. The questionnaire was divided into two parts. The first part includes sociodemographic data like age, gender, and marital status. The second part includes questions to assess: (1) knowledge and beliefs about causes and aggravating factors, (2) knowledge and beliefs about treatment, and (3) the perceived psychological effects of acne. Questionnaire was validated and modified in the light of pilot study. The questionnaire was reviewed by 2 faculty, one of whom has Saudi Arabian slang; revised questionnaires were compared and necessary modifications were made before finally approved by the reviewers. The questionnaire was then reviewed by researchers again, one of whom has Saudi Arabian slang before and after pilot study with minor linguistic modifications of some confusing words. The participants were approached in their clinics (male and female dermatology clinics). The questionnaires were distributed and explained to them after obtaining their verbal consent. Questionnaires were collected after being completed. A pilot study was conducted on 38 patients—different from the target group—to check the understanding and clarity of the questionnaire. Based on the results, some linguistic modifications of questions were made to avoid confusion about questions and make easier understanding and interpretation by participants. The data were entered and analyzed in a personal computer using statistical package for social sciences (SPSS) software version 16. Data were presented using descriptive statistics in the form of frequencies and percentages for qualitative variables and mean and standard deviation (SD) for quantitative variables. Chi-square test was used as appropriate to determine association. The level of statistical significance was set to be less than 0.05. The study was approved by the Ethical Committee of Postgraduate Saudi Board Program, Eastern Province. Verbal consent was obtained from the participants after explaining the objectives of the study to them. All questionnaires were anonymous, and collected data were kept confidential and not used except for the study purpose.

## 3. Results

In this study, 200 questionnaires were distributed, 180 acne patient completed the questionnaire, and 20 patients were excluded (18 patients of them did not complete the questionnaire and 2 were non-Saudi). Males accounted for 40% of the sample and females were 60%.

Table 1 illustrates sociodemographic characteristics of study population while Table 2 showed assessment of knowledge and believes about causes and aggravating factors among acne patients/study populations. Factors affecting total knowledge score about causes and aggravating factors of acne among study sample are illustrated in Table 3 while Table 4 shows perceived stress due to acne in study population according to their gender. Self-reported social effects of acne among study group are summarized in Table 5.

## 4. Discussion

### 4.1. Knowledge, Beliefs, and Misconceptions about Causes and Aggravating Factors

Thirty-two percent of our patients believed that acne is inherited or having genetic factors. These results were better than other studies; for example, in Poli et al. study, 25.2% perceived acne to be inherited from parents [30] while it was 18% of Tallab study sample [8]. Food items in general were considered as causes and/or aggravating factors of acne. Fatty food, chocolate, potato chips and spicy food were considered a cause of acne in 53.9%, 79.4%, 53.9%, and 29.4% of the sample, respectively. In Poli et al. study fatty food, chocolate, and snacks were thought to exacerbate acne by 62% and 45% of their sample, respectively [30]. In Al-Hoqail study 79% of acne patients sample believed that acne is related to diet [7].

Tension was believed to be related to acne by 65.6% of our patients. Almost the same result was found in Tallab study (65% of his sample) [8]. In Al-Hoqail study and Amado et al. study 80% and 71% of acne patients, respectively, believed that acne is related to stress [7, 31]. Cosmetic products were believed to aggravate acne according to 53.3% of our sample which is similar to Poli et al. study in which 58% of the respondents believed that cosmetics are aggravating acne [30]. Fifty-four percent of our sample believed that menses aggravate acne, which is similar to Poli study results (55% thought that menses affect acne adversely) [30] and comparable to Stoll et al. study (44% of their sample experienced premenstrual flares of their acne) [32]. Self -ygiene was believed by about two-thirds (67.8%) of our patients to be related to acne in contrast with Poli et al. study where 40% of responders believed that not washing is an acne aggravating factor [30].

Regarding general knowledge score, 41.7% of our study population showed good knowledge in contrast to study of Brajac et al. (2004), where only 11% have the overall score of correct answers [11]. There was significant correlation between total knowledge score and both age (where increasing age was associated with increasing total knowledge score (*P* = 0.012) and gender (where total knowledge score of females was better than males (*P* = 0.031).

These study results showed that poor knowledge, false beliefs, and many misconceptions are prevalent among Saudi acne patients in a way comparable to previous studies in other populations which include acne patients and/or normal populations of different cultures. This may reflect deficient acne patient education during their follow-up in their dermatology clinics.

### 4.2. Psychosocial Effects

Perceived stress was self-reported by 98 patients, that is, 54.4% of total sample. There was statistically significant correlation of self-reported being stressed due to acne with gender, (40.3% and 63.9% of males and females. resp. (*P* = 0.001)). This is expected since females are more health conscious and sensitive regarding their skin and their health seeking behavior to reflect this consciousness. The effect of acne on school performance was reported by 13.3% compared to only 6.4% of Do et al. study sample [24]. This is in contrast to Al-Hoqail study results, where 39% of his sample reported affected school performance due to acne [7]. Work performance was thought to be affected by 10.6% of our sample that is contrary to Al-Hoqail study in which 39% of his sample felt an affected work achievement [7], while spouse relationship, marriage willingness, and affected friendship relations were thought to be affected by 21.1%, 30.6%, and 17.2% % of our sample, respectively. These results were different from Al-Hoqail study results where it was 46%, 56%, 46%, respectively [7]. Differences from Al-Hoqail study may reflect difference in study populations which consisted in Al-Hoqail study of high school and college students in the central region of Saudi Arabia.

### 4.3. Treatment Seeking Behavior

Regarding seeking medical advice, twenty-two percent, 16.7%, 23.9%, and 37.8% of total sample visited their doctors within 3 months, 3–6 months, and 6–12 months and after 1 year from symptoms appearance, respectively. Similar results were found in Poli et al. study (2011), twenty-two percent, 14.2%, 12.4%, and 49.6% of their sample, but their sample consisted of acne patients and others who never had acne [30], in contrast to Al Robaee study (2005) in which, majority of his sample (40.3%) sought medical advice in the first three months [1]. Different result was found in Tallab study (2004), where the vast majority of his sample (76.2%) started more than one year [8], which is consistent with Tan et al. study (2001) in which 74% of patients waited more than 1 year before seeking medical attention for acne [15]. This variability in treatment seeking behavior may be related to underlying deficiencies in knowledge and believes about acne both among general population and among acne patients. Whether acne needs to be treated once noticed or discovered, 75% of our sample agreed on that. In our study 60% (109 patients) agreed that acne does need to be treated by physicians, which is consistent with Poli et al. study, where 70.9% subjects in their study believed that acne should be treated by physicians [30]. Forty-seven percent of our study believed that treating acne by doctors requires Long-term follow-up which is compared to only 26% in Al-Robaee study [1]. The majority of patients (87.2%) in our study believed that the outcome of the treatment by physicians is guaranteed, and 90.6% considered it safe.

## 5. Conclusion and Recommendations

These study results showed that poor knowledge, false beliefs, and many misconceptions are prevalent among Saudi acne patients in a way comparable to the previous studies in other populations and cultures. This in spite of the fact that our study population consisted only of acne patients followed up in dermatology clinics. Seeking medical advice behavior and expectation from treatment modalities among acne patients in this study are also similar to other studies and appear to reflect the poor knowledge and misconceptions about the disease. More effort for health education in general and selective patient education in particular is needed to improve patients' knowledge about acne and its modalities of treatment and to encourage early medical consultation behavior and improve patient adherence to treatment. Considering psychological effect, it appears to be high as it has been proved in other cultures and needs always to be considered and addressed early in the course of patient management.

## Figures and Tables

**Table 1:** Sociodemographic characteristics of study population.

Variable	Frequency (total sample number = 180)	
	No.	%
Age		
≤14 years old males	4	2.2%
≤14 years old females	15	8.4%
14–21 years old males	52	28.9%
14–21 years old females	60	33.3%
>21 years old males	24	13.3%
>21 years old females	25	13.9%
Gender		
Males	72	40%
Females	108	60%
Marital status		
Single	150	83.3%
Married	28	15.5%
Divorced	1	0.6%
Widow (er)	1	0.6%
Education		
Illiterate	5	2.8%
Primary school	2	1.1%
Intermediate school	29	16.1%
Secondary school	95	52.8%
Bachelor or more	49	27.2%
Occupation		
Student	101	56.1%
Governmental job	15	8.3%
Nongovernmental	20	11.1%
Housewife	16	8.9%
Jobless	28	15.6%
Income		
<5000 Saudi Riyals	54	30%
5000–10000 Saudi Riyals	82	45.6%
>10000 Saudi Riyals	44	24.4%

**Table 2:** Knowledge about causes and aggravating factors among acne patients.

Factors	Yes		No		Do not know	
	No.	%	No.	%	No.
Inheritance (genetics)	57	31.7	81	45	42	23.3
Consuming fatty food*	97	53.9	53	29.4	30	16.7
Consuming chocolate*	143	79.4	27	15	10	5.6
Consuming spicy food*	53	29.4	83	46.1	44	24.4
Consuming potato chips*	97	53.9	52	28.9	31	17.2
Obesity*	61	33.9	67	37.2	52	28.9
Poor hygiene*	122	67.8	37	20.6	21	11.7
Tension	118	65.6	32	17.8	30	16.7
Using cosmetics	96	53.3	33	18.3	51	28.3
Menses	98	54.4	23	12.8	59	32.8
Exposure to sun*	63	35	62	34.4	55	30.6
Contagious*	56	31.1	68	37.8	56	31.1

*Indicate wrong answer.

**Table 3:** Factors affecting total knowledge score about causes and aggravating factors of acne among study sample.

	Poor		Good		Total		*P* value
	No.	%	No.	%			
Knowledge versus age							
Childhood	14	73.7%	5	26.3%	19	100.0	*P* = 0.012
Teenagers	69	61.6%	43	38.4%	112	100.0	
Adulthood	22	44.9%	27	55.1%	49	100.0	
** **Total	**105**	**58.3%**	**75**	**41.7%**	**180**	**100.0**	
Knowledge versus gender							
Males	49	68.1	23	31.9	72	100.0	*P* = 0.031
Females	56	51.9	48.1	48.1	108	100.0	
** **Total	**105**	**58.3**	**75**	**41.7**	**180**	**100.0**	
Knowledge versus marital status							
Single	90	60	60	40	150	100.0	*P* = 0.357
Married	13	46.4	15	53.6	15	100.0	
Divorced	1	100	0	0	1	100.0	
Widow (er)	1	100	0	0	1	100.0	
** **Total	**105**	**100**	**75**	**41.7**	**180**	**100.0**	
Knowledge versus education							
Illiterate	4	80	1	20	5	100.0	*P* = 0.297
Primary school	2	100	0	0	2	100.0	
Intermediate	20	69	9	31	29	100.0	
Secondary	54	56.8	41	43.2	95	100.0	
Bachelor and more	25	51	24	49	49	100.0	
** **Total	**105**	**58.3**	**75**	**41**	**180**	**100.0**	
Knowledge versus income							
<5000 RS	33	61.1%	21	38.9%	54	100.0	*P* = 0.258
5000–10000** **RS	51	62.2%	31	37.8%	82	100.0	
>10000 RS count	21	47.7%	23	52.3%	44	100.0	

**Table 4:** Perceived stress due to acne in patients according to gender.

	Not affected		Affected		Total		*P* value
	No.	%	No.	%			
Male	43	59.7%	29	40.3%	72	100%	*P* = 0.001
Female	39	36.1%	69	63.9%	108	100%	

**Table 5:** Self-reported social effects of acne.

Life activity	Total (*N* = 180)	%
School performance	24	13.3%
Work performance	19	10.6%
Spouse relationship	38	21.1%
Willing to get married	55	30.6%
Friendship affected	31	17.2%
